# Malaria control in the African Region: perceptions and viewspoints on proceedings of the Africa Leaders Malaria Alliance (ALMA)

**DOI:** 10.1186/1753-6561-5-S5-S3

**Published:** 2011-06-13

**Authors:** Luis Gomes Sambo, Georges Ki-Zerbo, Joses Muthuri Kirigia

**Affiliations:** 1 World Health Organization, Regional Office for Africa, B.P. 06, Brazzaville, Congo

## Abstract

**Background:**

In 2009 a total of 153,408 malaria deaths were reported in Africa. Eleven countries showed a reduction of more than 50% in either confirmed malaria cases or malaria admissions and deaths in recent years. However, many African countries are not on track to achieve the malaria component of the Millennium Development Goal (MDG) 6. The African Leaders Malaria Alliance (ALMA) working session at the 15^th^ African Union Summit discussed the bottlenecks to achieving MDG 6 (specifically halting and beginning to reverse the incidence of malaria by 2015), success factors, and what countries needed to do to accelerate achievement of the MDG. The purpose of this article is to reflect on the proceedings of the ALMA working session.

**Methods:**

Working methods of the session included speeches and statements by invited speakers and high-level panel discussions.

**Discussion:**

The main bottlenecks identified related to the capacity of the health systems to deliver quality care and accessibility issues; need for strong, decentralized malaria-control programmes with linkages with other health and development sectors, the civil society and private sector entities; benefits of co-implementation of malaria control programmes with child survival or other public health interventions; systematic application of integrated promotive, preventive, diagnostic and case management interventions with full community participation; adapting approaches to local political, socio-cultural and administrative environments.

The following prerequisites for success were identified: a clear vision and effective leadership of national malaria control programmes; high level political commitment to ensure adequate capacity in expertise, skill mix and number of managers, technicians and service providers; national ownership, intersectoral collaboration and accountability, as well as strong civil society and private sector involvement; functional epidemiological surveillance systems; and levering of African Union and regional economic communities to address the cross-border dimension of malaria control.

It was agreed that countries needed to secure adequate domestic and external funding for sustained commitment to malaria elimination; strengthen national malaria control programmes in the context of broader health system strengthening; ensure free access to long-lasting insecticide treated nets and malaria diagnosis and treatment for vulnerable groups; strengthen human resource capacity at central, district and community levels; and establish strong logistics, information and surveillance systems.

**Conclusion:**

It is critically important for countries to have a clear vision and strategy for malaria elimination; effective leadership of national malaria control programmes; draw lessons from other African countries that have succeeded to dramatically reduce the burden of malaria; and sustain funding and ongoing interventions.

## Background

Dr Gro Harlem Brundtland, the then Director-General of the World Health Organization (WHO), identified Roll Back Malaria (RBM) as a priority project of the renewed WHO and established it as such on 23 July 1998 [1]. The goal of RBM was to contribute to strengthen national health systems working to significantly reduce the burden of disease associated with malaria through better access of poor people to a range of effective antimalaria interventions. The global partnership for RBM was established at a meeting in Geneva, 8–9 December 1998. The membership is drawn from representatives of countries affected by malaria, organizations of the United Nations system, bilateral development agencies, development banks, nongovernmental organizations and the private sector. RBM is committed to a common purpose, ways of working and outcomes.

In September 2000 the UN added efforts to fight malaria to the Millennium Development Goals (MDGs) [2]. On 25 April 2000 African Heads of State and Government at the African Summit on Roll Back Malaria in Abuja, Nigeria, signed the Abuja Declaration and Plan of Action with a firm commitment to halve malaria mortality for Africa’s people by 2010 [3]. The African leaders also declared 25th April of each year as “Africa Malaria Day”. The Assembly of the African Union adopted a declaration on Malaria, HIV/AIDS, Tuberculosis and Other Related Infectious Diseases at its second ordinary session held in Maputo, Mozambique, 10–12 July 2003 [4]. In May 2005, the World Health Assembly endorsed the target of halving the malaria burden by the end of 2010 from the 2000 levels [5].

At the Abuja Summit in 2006 African Union Heads of State and Government called for universal access to HIV/AIDS, tuberculosis and malaria services by a united Africa by 2010 and for a long-term vision on malaria elimination [6]. In May 2007 the World Health Assembly approved resolution WHA60.18 establishing the World Malaria Day and urging all Member States to apply a broad range of actions to scale up malaria control programmes and in particular to deploy recommended artemisinin-based combination therapies and progressively withdraw from the market oral artemisinin-based monotherapies [7].

In 2008 the Global Malaria Action Plan was developed, and the malaria endemic countries developed road maps to scale up cost-effective interventions towards achieving the MDGs [8]. The same year the UN Secretary General put forward a bold vision aimed at stopping malaria deaths by ensuring universal coverage by the end of 2010 [9]. This initiative was geared at (i) offering indoor residue spraying and bed nets treated with long-lasting insecticide to all people at risk, especially women and children in Africa, (ii) ensuring that all public health facilities had access to effective malaria treatment and diagnosis, (iii) providing ways to train and retain community health workers dealing with malaria, and (iv) encouraging research and development for longer term efforts to control, eliminate and eradicate malaria [9].

In September 2009 the 59th WHO Regional Committee for Africa adopted a resolution on Accelerated Malaria Control, Towards Elimination in the African Region, which calls for, among other actions, integration of malaria control in poverty reduction strategies and all national health and development plans and mobilization of local resources for sustainable implementation of the strategies [10].

On 23 September 2009 His Excellency President Jakaya Kikwete of Tanzania, Past Chairman of the African Union, launched the African Leaders Malaria Alliance (ALMA) during the Sixty-fourth Session of the United Nations General Assembly [11]. The purpose of ALMA is to provide African leaders with a high-level forum to ensure efficient procurement, distribution and utilization of malaria control tools; facilitate the sharing of effective malaria control practices; and ensure that malaria remains high on the global policy agenda. ALMA leaders subsequently met in February 2010 in Addis Ababa, Ethiopia, to further refine their vision and strategic agenda [12].

The ALMA working session at the 15th African Union Summit in Kampala was held 25–26 July 2010 in the context of calls for increasing efforts to accelerate progress towards MDGs 4 (reduce child mortality), 5 (improve maternal health and reduce maternal mortality) and 6 (combat HIV/AIDS, malaria and other diseases in particular through halting and beginning to reverse the incidence of malaria by 2015) and was in line with the theme of the Summit, ‘Maternal, Infant and Child Health and Development in Africa’. The session agenda was also well aligned with the issues discussed at the previous satellite session on health financing.

The purpose of this article is to reflect on the proceedings of the ALMA working session.

## Methods

The session included speeches and statements by invited speakers and high-level panel discussions. It was chaired by His Excellency Jakaya Mrisho Kikwete, President of the United Republic of Tanzania. The panel comprised the host of the African Union Summit, President Yoweri Museveni of Uganda, President Robert Mugabe of Zimbabwe and ministers of health and officials from African Union Member States. Invited speakers included Mr Raymond G. Chambers, UN Secretary General's Special Envoy for Malaria; Mrs Asha-Rose Migiro, UN Deputy Secretary-General; Mrs. Obiageli Ezekwesili, World Bank Vice President for Africa; Professor Michel Kazatchkine, Executive Director of the Global Fund to Fight Aids, Tuberculosis, and Malaria (GFATM); Mr Kapembwa Simbao, Minister of Health of Zambia and Chairman of the Roll Back Malaria Partnership Board; Dr Luis Gomes Sambo, WHO Regional Director for Africa; and Mrs Johannah-Joy Phumaphi, Executive Secretary of ALMA.

## Summary of presentations

### Statement by His Excellency President Jakaya Mrisho Kikwete

In his opening remarks, President Jakaya Mrisho Kikwete, stated [13]: “I see this meeting of ours today as the watershed in our efforts and quest to control and eliminate malaria in our respective countries and therefore in the entire African continent. By establishing ALMA today, we are now creating a critical forum and mechanism for advocacy, action, and follow-up on the implementation of these noble goals.” He indicated that mosquito net coverage in 20 African countries was at least five times higher in 2010 than in 2000, leading to significantly fewer cases of the disease and death. He apprised that on the Tanzanian island of Zanzibar, the combination of high mosquito net coverage, ACT availability and indoor residue spraying had reduced malaria deaths by over 90%. More importantly, under-five deaths from all causes fell by almost three-fifths.

President Kikwete underlined two strategic goals: (i) to achieve universal mosquito net coverage by the end of 2010 and (ii) to eliminate preventable malaria deaths by 2015 by scaling up coverage of all other available interventions (including access to rapid diagnostic tests, treatment with artemisinin-based combination therapy, intermittent preventive treatment and indoor residual spraying).

He acknowledged that these were audacious goals, but with more than 680,000 African children dying each year from malaria, audacity was a moral imperative. He strongly advocated for malaria control as a step towards achieving MDGs 4 (child mortality) and 5 (maternal health). As convener of ALMA, President Kikwete noted the tremendous contribution of the Global Fund to Fight AIDS, Tuberculosis and Malaria (GFATM) and the World Bank in helping to finance malaria control in Africa. He expressed ALMA support for the full replenishment of funding to both GFATM and the International Development Association of the World Bank.

Finally, President Kikwete called upon African leaders, communities and partners to make a final push to reach universal coverage of interventions against malaria.

### Statement by Mr Raymond G. Chambers, UN Special Envoy for Malaria

The UN Secretary General Special Envoy for Malaria focused his statement on the need to achieve universal coverage with essential interventions to end preventable deaths due to malaria [14]. He highlighted the success in procuring more than 200 million nets, including through bulk purchasing. He reported on the MDG meeting and stated that African leadership, represented by ALMA, was a model that could be emulated to help accelerate progress for the other health related MDGs. Mr Chambers advocated for the remaining countries to join ALMA and invited the Heads of States and Government to the September 2010 UN General Assembly MDG event.

### Statement by Dr Asha-Rose Migiro, UN Deputy Secretary-General

The UN Deputy Secretary-General, addressing the AU leaders, indicated that the fight against malaria was boosting women’s and children’s health [15]. She applauded the successes reported in several African countries and called upon the leadership of African heads of states: “If you continue to see malaria control as an integral part of reaching the Millennium Development Goals … of building strong health systems … of improving your people’s well-being … then the success we have seen to date will continue, and grow.” The UN Deputy Secretary-General acknowledged that the fight against malaria would be long, but she added: “We are on the road to success. With perseverance, we will win.”

### Statement by Mrs Obiageli Ezekwesili, Vice President of the World Bank for Africa

The World Bank Vice President for Africa indicated that the World Bank was making investments in malaria control that would have returns both in terms of lives saved and well beyond the health sector [16]. Those investments are based on the evidence that malaria control contributes to health and development in Africa. Dr Ezekwesili expressed the World Bank’s commitment to strengthening support to ALMA as it deploys innovative approaches to end deaths from malaria.

### Statement by Professor Michel Kazatchkine, Executive Director of GFATM

The Executive Director of GFATM presented recent evidence on the impact of GFATM supported national programmes in saving lives and contributing to health systems and development [17]. He strongly advocated for the support of recipient countries, bilateral and multilateral donors and the UN for the replenishment of the fund in order to sustain the gains being made in disease control and improvements in maternal and child health.

### Statement by Dr Luis Gomes Sambo, Regional Director, WHO Regional Office for Africa

Dr Luis G. Sambo informed the session that the African Region accounted for 85% of malaria episodes and 89% of malaria deaths worldwide [18]. He reminded the audience that approximately one in every five childhood deaths was attributable to the effects of malaria and that the disease could result in miscarriage, low birth weight and maternal death rates ranging from 10 to 50%. Malaria impacts negatively on MDGs 4 and 5. He underscored the fact that the economic and health losses associated with malaria were significant.

Dr Sambo reported that over the last decade, African countries had scaled up proven malaria interventions, which include vector control using long-lasting insecticide-treated nets [19,20], indoor residue spraying [21], intermittent preventive treatment in pregnancy [22] and prompt and effective diagnosis and treatment [23].

Dr Sambo highlighted the encouraging achievements of Botswana, Cape Verde, Eritrea, Namibia, Rwanda, Sao Tome and Principe, South Africa, Swaziland, Zambia and Zanzibar (Tanzania) in reducing the number of malaria cases by at least 50% between 2000 and 2008 [24]. This success is associated with strong leadership from heads of states and community leaders. He encouraged the remaining countries to scale up the coverage of interventions to reach universal coverage.

He informed the meeting that some countries were contemplating the transition from sustained control to elimination of malaria owing to their very low malaria transmission levels and based on robust epidemiological evidence and feasibility assessments. Governments contemplating the pursuit of malaria elimination will need to secure adequate domestic and external funding for sustained commitment; strengthen national malaria control programmes in the context of broader health systems strengthening; strengthen human resource capacity at central, district and community levels; establish strong logistic, information and surveillance systems as well as early detection mechanisms for response to malaria epidemics and complex emergencies; and forge effective cross-border and trans national malaria control collaborative initiatives.

According to Dr Sambo, the use of oral artemisinin monotherapy was a potential driver in emergence of resistant malaria parasites. In May 2007 the World Health Assembly adopted Resolution WHA60.18, which urged Member States to deploy recommended artemisinin-based combination therapies (ACTs) and withdraw oral artemisinin-based monotherapies. He informed the meeting that by July 2010, 30 countries in the African Region had taken regulatory measures to withdraw oral artemisinin-based monotherapies. He urged the remaining countries to urgently develop similar policies to stop the marketing of oral artemisinin-based monotherapies.

Dr Sambo cited a study that estimated at US$ 3 billion per year Africa’s need for global resources to attain international malaria control goals. Financial support to African countries for prevention and control of malaria totals approximately US$1.6 billion per year and comes from GFATM, the US President’s Malaria Initiative (PMI), the World Bank Booster Programme for Malaria Control in Africa, the United Kingdom Department for International Development (DFID), Bill and Melinda Gates Foundation and other agencies and foundations. He indicated that based on the cost of an essential package of interventions, this support represents 53% of the estimated financial resources needed to reach global malaria control targets [25]. He appealed to external partners to support countries to cover the funding gap of approximately US$1.4 billion per year for universal coverage of key malaria interventions and to boost capabilities for attaining the MDG targets.

He concluded by appealing to the Heads of State to foster leadership for improved intersectoral collaboration to eliminate malaria as a public health problem in Africa; increase domestic funding for malaria control and accelerate progress towards MDGs 4, 5 and 6; support viable initiatives for local production of quality medicines and other commodities; eliminate taxes and tariffs on antimalarials and other public health essential commodities; and ensure free access to malaria diagnosis and treatment for vulnerable groups such as children and pregnant women, in addition to universal access to long-lasting insecticide treated nets.

### Statement by Mrs Johannah-Joy Phumaphi, ALMA Executive Secretary

Mrs Johannah-Joy Phumaphi reported on the ALMA call for immediate acceleration of procurement, shipment and distribution of malaria commodities [26]. She explained how the ALMA tender in collaboration with UNICEF, WHO and ministers of health is building capacity in participating countries. She identified the gap between ownership of long-lasting insecticide treated nets and their effective use as a critical barrier to be addressed by malaria programmes. She emphasized that ALMA would continue to work with countries and partners to ensure that communities were empowered to hang, use and maintain the nets properly, and to disseminate best practices. She reported the commitment of African ministers of heath, which was reiterated at the Roll Back Malaria Board’s Special Session during the African Union Summit.

Mrs Johannah-Joy Phumaphi expressed concern that the high tariffs on commodities, such as bed nets and antimalarial drugs, make them expensive and hamper access. She cited the latest data that indicated that only five African countries had completely eliminated these tariffs.

## Discussion

In his remarks to ALMA, Mr Kapembwa Simbao, Minister of Health of Zambia [27], expressed his support for the ‘Actions for Accelerated Achievement of Maternal, Newborn and Child Health and Development in Africa’ adopted by the AU to ensure achievement of the health MDGs 4, 5 and 6 by 2015 [28].

Zimbabwe, Zambia, Rwanda, Nigeria, Mozambique, Kenya and Mauritius presented their experiences in scaling up of malaria control interventions, cross-border collaboration and malaria elimination challenges. These experiences revealed the link between malaria control and maternal and child health. The debate provided insights on the importance of implementing comprehensive programmes, including community involvement, the need to build adequate capacity for vector control, and the use of rapid diagnostic tests. The role of nongovernmental organizations (NGOs) and public-private partnerships was also covered. The innovative approaches highlighted included involvement of school children and teachers in Mozambique, building capacity of village health workers in Zimbabwe, fostering cross-border collaboration among Mozambique, Swaziland and South Africa, and establishing model health centres in Kenya. Mauritius talked on its efforts to prevent reintroduction of malaria by using active surveillance and enforcing travel health regulations.

Cross-border collaboration is necessary for malaria control in Africa because of the large negative externalities involved. Elimination of malaria in one country will not be sustained if the neighbouring countries experience high transmission of malaria, as citizens mix freely along borders, re-introducing malaria through travel and migration. Cross-border mechanisms that could be leveraged for controlling malaria already exist in the African Region. There are two recent examples. First, on 18^th^ October 2010, the ministers of health from the republics of Benin, Cameroon, Central African Republic, Chad, Equatorial Guinea, Niger and Nigeria met in Abuja and signed the Abuja Commitment on Cross-Border Public Health Issues. The countries represented agreed to establish a joint initiative that permits multi-sector involvement in prevention and control of multiple public health problems in participating countries; promote communities-based initiatives on information, education and communication for disease prevention and control; promote cross-border initiatives in early detection of epidemics and improvement of water supply, sanitation and hygiene; advocate for operationalization of the African Public Health Emergency Fund to support emergency and cross-border public health activities; strengthen National Disease Surveillance Systems and share information on cross border health issues; establish National Agency for Drug Control and inter-country multi-disciplinary committees to monitor the quality of medicines and the circulation of counterfeit medicines across borders; provide (on request) technical and logistics support to any of the participating countries in the management of public health emergencies; and appoint focal persons to constitute a committee to follow-up recommendations made in the inter-ministerial meeting [29].

Second, on 18^th^ March 2011, the ministers of health of the Republic of Angola, Republic of Congo, Democratic Republic of Congo, Republic of Namibia and Republic of Zambia met in Lusaka and signed a Memorandum of Understanding (MOU) on Cross Border Public Health Issues. The MOU touches on progress towards achievement of health related MDGs; strengthening health systems to support cross border public health issues; prevention and control of cholera and other epidemic prone diseases; polio eradication; surveillance and international health regulations; fake and counterfeit medicines; and monitoring and evaluation of cross border public health issues. Such mechanisms can be used to mobilize domestic and external resources, design, implement, monitor and report on cross-border public health action against infectious diseases [30].

The country presentations echoed the sentiment of the high-level panel that the burden of malaria should be addressed as a priority health, social and developmental issue and no longer as a fatality threat for Africa. The need of tackling vector, parasite and host factors in the context of specific environmental and climatic conditions also was highlighted during the debate. Speakers at the session emphasized the necessity of multi-pronged approaches as the way forward to sustain the gains in reduction of malaria morbidity and mortality. There was agreement that the old but still valid notion of the pathogenic malaria complex must remain at the core of malaria control and elimination strategies [31].

The main bottlenecks identified by country representatives related to the capacity of the health systems to deliver quality care and to issues of ensuring universal access to diagnostic services and treatment. The participants unanimously agreed on the need for strong, decentralized malaria-control programmes with linkages with other health and development sectors, the civil society and private sector entities. Recent publications highlight the benefits of co-implementation of malaria control programmes with child survival or other public health interventions [10,32-34]. For example, on Bioko Island in Equatorial Guinea and in the Chongwe district area of Zambia, significant reduction of the malaria burden has had a major impact on overall child survival. The effect is both direct and indirect through systematic application of integrated promotive, preventive, diagnostic and case management interventions with full community participation. In Zambia, malaria related outpatient visits have reduced, and the reduction in malaria cases at health facilities has led to an increase in diagnoses of respiratory infections. These findings have implications for the management of non-malaria fevers in children under the age of five years. Similar data on cost-effectiveness and impact of quality malaria diagnostic and treatment are emerging in other countries including Kenya, Senegal and Gambia [32-38]. Successful experiences and best practices from Zanzibar (Tanzania), Ethiopia, Rwanda and Eritrea show that it is possible to scale up essential interventions in the context of existing programme structure while adapting approaches to local political, socio-cultural and administrative environments [39-43].

However, it is important to acknowledge the challenges that have to be overcome in ensuring universal access to malaria prevention and control services; and getting people to use those services once access has been assured. The first challenge is to develop sustainable health financing mechanisms that assure free access to diagnosis, treatment and insecticide treated nets for the poor. Such mechanisms may entail a combination of social health insurance and tax funded health services. In addition, health development partners can complement government efforts by paying social health insurance (or community health insurance) premiums on behalf of the poor. Furthermore, partners can reinforce national governments capacities for providing free access for the poor through general budget support. The second challenge draws attention to the fact that even with free access, utilization may be a problem because non-financial barriers to use of diagnostic services and medication can be significant [44]. The non-financial barriers (e.g. limited health-related knowledge and awareness, behavioural factors, cultural beliefs, geographic isolation) may be addressed by leveraging health promotion methods and approaches [45-47].

The success mentioned above has been made possible by a number of enabling factors. First, existence of a clear vision and effective leadership contributed to improvement in the governance, stewardship and success of national malaria control programmes. Second, high level commitment has ensured relatively adequate capacity in expertise, skill mix and number of managers, technicians and service providers, including for community interventions and case management. Third, national ownership, intersectoral collaboration and accountability, as well as strong civil society and private sector involvement, are important attributes of well-performing malaria control programmes. Fourth, successful programmes have the capacity to adjust to the rapidly changing malaria epidemiology. Fifth, several regional initiatives initiated with the support of AU and regional economic communities contributed in addressing the cross-border dimension of malaria control.

The panellist’s emphasized countries that had achieved significant reductions in the malaria burden required support to undertake programme assessments to ensure sustained control and evidence-based transition to elimination of the disease. This requires in-depth programme reviews, strengthening of capacity for surveillance monitoring and evaluation and intensification of cross-border collaboration. It will entail contribution of research and research based institutions in the collection, analysis and dissemination of information for better control, adjustments, and policy guidance.

The meeting was informed that the WHO regional framework for the acceleration of malaria control towards elimination and the guidelines on malaria elimination were available to guide the countries in planning, implementation, monitoring and evaluation of malaria programmes [48]. Moreover, WHO technical support is available to the African Union to design relevant policies and strategies, in particular the primary health care approach for strengthening district health systems [33,49]. Towards this endeavour, governments should address the critical challenges including:

• Securing adequate domestic and external funding for sustained commitment to malaria elimination;

• Strengthening national malaria control programmes in the context of strengthening the broader health system;

• Ensuring free access to malaria diagnosis and treatment for vulnerable groups such as children and pregnant women in addition to universal access to long-lasting insecticide treated nets;

• Strengthening human resource capacity at central, district and community levels;

• Establishing strong logistics, information and surveillance systems as well as early detection mechanisms for response to malaria epidemics and other public health threats.

## Conclusions

A number of conclusions emerged from the discussions. First, ALMA working sessions at the African Union Summit were critically important for sustaining the focus on accelerated malaria control and elimination vision and strategy in support of actions at the country level. Second, there is need for documenting and sharing lessons learnt from African countries that have succeeded to dramatically reduce the burden of malaria. Third, the achievements in some countries clearly indicate that the Abuja and MDG malaria targets are achievable if funding and ongoing interventions are sustained. Lastly, there was a strong indication that the next MDG summits will also offer a forum for strategic projections and consolidation of financing and implementation pledges [50,51].

## List of abbreviations used

ACTS: Artemisinin-based Combination Therapies; ALMA: African Leaders Malaria Alliance; GFATM: Global Fund to Fight AIDS, Tuberculosis and Malaria; MDG: Millennium Development Goal; NGO: Non-governmental Organization; UN: United Nations Organization; WHO: World Health Organization.

## Competing interests

The authors declare that they have no competing interests.
